# Mechanical Ventilator Parameter Estimation for Lung Health through Machine Learning

**DOI:** 10.3390/bioengineering8050060

**Published:** 2021-05-07

**Authors:** Sanjay Sarma Oruganti Venkata, Amie Koenig, Ramana M. Pidaparti

**Affiliations:** 1 College of Engineering, University of Georgia, Athens, GA 30602, USA; sanjaysarmaov@uga.edu; 2 College of Veterinary Medicine, University of Georgia, Athens, GA 30602, USA; akoenig@uga.edu

**Keywords:** mechanical ventilation, respiratory health, machine learning, artificial neural networks, particle swarm optimization

## Abstract

Patients whose lungs are compromised due to various respiratory health concerns require mechanical ventilation for support in breathing. Different mechanical ventilation settings are selected depending on the patient’s lung condition, and the selection of these parameters depends on the observed patient response and experience of the clinicians involved. To support this decision-making process for clinicians, good prediction models are always beneficial in improving the setting accuracy, reducing treatment error, and quickly weaning patients off the ventilation support. In this study, we developed a machine learning model for estimation of the mechanical ventilation parameters for lung health. The model is based on inverse mapping of artificial neural networks with the Graded Particle Swarm Optimizer. In this new variant, we introduced grouping and hierarchy in the swarm in addition to the general rules of particle swarm optimization to further improve its prediction performance of the mechanical ventilation parameters. The machine learning model was trained and tested using clinical data from canine and feline patients at the University of Georgia College of Veterinary Medicine. Our model successfully generated a range of parameter values for the mechanical ventilation applied on test data, with the average prediction values over multiple trials close to the target values. Overall, the developed machine learning model should be able to predict the mechanical ventilation settings for various respiratory conditions for patient’s survival once the relevant data are available.

## 1. Introduction

In a healthy person, spontaneous breaths are normally generated when respiratory muscles contract, pull the pleura, and create a negative intrapleural pressure, allowing airflow into the lungs (negative pressure ventilation). During this process, oxygen is exchanged for ${\mathrm{CO}}_{2}$ in the alveoli. Patients suffering from respiratory failures, such as from congestive heart failure, COPD (chronic obstructive pulmonary disease), pneumonia, ARDS, and recently COVID-19 [1,2,3,4,5], or patients with ventilatory failures, such as those resulting from central or peripheral neurologic or muscular dysfunction, may require external mechanical ventilatory support.

COVID-19 is a rapidly changing condition, and very little has been researched so far in 2021 on the practice of deciding ventilation parameters for different patients and conditions [3]. Additionally, it is also understood to be challenging to determine when to intubate and mechanically ventilate a hospitalized patient [2,5]. Many discrepancies have been reported in COVID-19 ICU and ventilator management studies due to regional and temporal variations [4]. This emphasizes the need for comprehensive research focused mainly on developing mechanical ventilator parameter tuning methods accommodating the patient response.

In most cases, a compromise in respiratory process would either lead to hypercapnia (hypoventilation), with elevated ${\mathrm{CO}}_{2}$ in the blood, or hypoxemia, with abnormally low blood oxygen level. External support to these patients is provided through positive mechanical ventilation until their condition improves and they are weaned off the machines [6]. The most common modes of mechanical ventilation used are pressure and volume controlled [7]. In volume-controlled ventilation (VCV), tidal volume is set at a constant value, while inspiratory pressure is a dependent variable. In VCV, inspiratory flow and waveform parameters are set by the clinicians, and the system responds to reduced compliance and active inhalation by increasing pressure, which might also increase the risk for lung injuries.

Similarly, in pressure-controlled ventilation (PCV), the pressure is the set variable, and tidal volume is a dependent variable. In order to mitigate the risk, running the ventilator in a PCV mode can limit the maximum airway pressure but can alter the tidal flow volume and waveform patterns, reducing the effectiveness of ventilation. Campbell et al. [8] presented a comparison between VCV and PCV modes and recommended using hybrid dual-control modes. Other ventilation modes include synchronized intermittent mandatory ventilation (SIMV), where the patient-initiated breath is detected, and a required preset volume or pressure is delivered. This mode helps patients to wean off the ventilator by allowing spontaneous breaths in between ventilator-administered mandatory breaths. Similar to SIMV, pressure support ventilation (PSV) also provides support by delivering a preset pressure to the patient for every patient-initiated breath that exceeds the inspiratory trigger value. The PSV pressure can be different from the mandatory breath pressure and vary with the patient’s needs.

Modern mechanical ventilators allow clinicians to adjust a variety of parameters along with the mode of operation according to patient’s condition [9]. Respiratory rate (RR) parameters regulate the mandatory breathing rate per minute (also called SetRR), and ActRR is the sum of mandatory breaths and additional patient-initiated breaths. The percentage of inspired oxygen can be set from room air (∼21%) to 100%. The SetP parameter regulates the air pressure to be delivered to the patient in PCV, and a peak value can also be set. The total pressure, peak inspiratory pressure (PIP), is the sum of the airway pressures PEEP and SetP. In addition, a set constant positive end-expiratory pressure (PEEP) can be provided by the ventilator during exhalation to help prevent alveolar collapse between breaths.

The patient’s observed heart rate, blood pressure, temperature, blood oxygen level (${\mathrm{SpO}}_{2}$); the concentration of carbon dioxide at the end of the exhaled breath (${\mathrm{EtCO}}_{2}$); and inspiratory tidal volume ($V_{ti}$) are among the commonly observed response parameters. In addition to these, the dynamic compliance ($C_{dyn}$) parameter measures the distensibility of lungs [9], which is computed by,

(1) $$C_{dyn}=\frac{V_{ti}}{PIP-PEEP}$$

Every patient is different, and treating them will require a different degree of understanding and control of the ventilator settings. The proper selection of ventilation modes and parameters is crucial for optimal treatment, a complex process requiring a high level of expertise from the clinicians. The challenge is in creating a specific model with a diffuse set of patients and conditions [3], and evidence-based practice can support accurate parameter estimations for optimal, errorless, and low-cost treatments. Ervin et al. [10] review these evidence-based approaches for optimizing invasive mechanical ventilation use (IMV) for acute respiratory distress syndrome (ARDS).

Recently, the use of AI and machine learning-based decision support approaches combined with evidence-based practices has grown, leading to better patient management and treatment [11,12,13]. Many models applying artificial neural networks (ANNs) have been proposed in establishing a relationship between the MV parameters and patient outcomes. Akbulut et al. [14] proposed a model for estimating frequency, tidal volume, and ${\mathrm{FiO}}_{2}$ outputs of a ventilator and a classification model capable of deciding the pressure and volume control modes using artificial neural networks (ANN). A disease detection Bayesian forecast model was applied to the data to detect disease type before applying ANN in the second-stage system. Their proposed ANN model uses the diagnosed disease type from stage one (Bayesian forecast), core body temperature, heart rate, blood pressure, PEEP, ${\mathrm{SpO}}_{2}$, pH, ${\mathrm{EtCO}}_{2}$, and bicarbonate data as input features. Frequency, $V_{ti}$, ${\mathrm{FiO}}_{2}$, and the pressure/volume support value are the predictions. The proposed model showed accuracies of 95% in predicting ${\mathrm{FiO}}_{2}$ values in real-time.

A previous study in this area by Nelson et al. focuses on a fuzzy-based control method Nelson et al. [15] uses the patient and heuristics data obtained from clinician’s experience. These data are combined with control principles to adjust the respiratory rates. The designed fuzzy logic controllers performed well on simulated-patient scenarios emulating the decisions of an experienced clinician. Tehrani et al. [16] presented a novel model for tuning the parameters for optimal blood gas regulation, minimizing the breathing work rate, and increasing the weaning rate of the ventilators.

Further, Kwong et al. [17] reviewed various computational intelligence and machine learning techniques applied on predicting and guiding the weaning process in patients. They argued that model-based systems are prone to sub-optimal outcomes due to their dependence on assumptions and, hence, prescribed machine learning-based models for ventilator control. The study further investigated the performance of nine different models proposed in the literature and ranked them based on a set of appraisal points. Their study included Giraldo et al.’s [18] work on Support Vector Machines (SVM) and ANN classification [19] approaches for weaning trials in patients. Along the same lines, we introduce an inverse mapping technique on ANNs for parameter estimation with the Graded Particle Swarm Optimizer (GPSO) in the feedback loop.

The remainder of this paper is organized as follows: In Section 2, we present our machine learning model based on inverse mapping of neural networks and introduce the GPSO algorithm followed by an outline of our prediction model. In Section 3, we introduce the data utilized for our analysis, followed by pruning and ANN training methods. Section 4, presents the analysis results, followed by conclusions in Section 6.

## 2. Machine Learning Model

### 2.1. Inverse Mapping of Artificial Neural Networks

**Figure 1:**
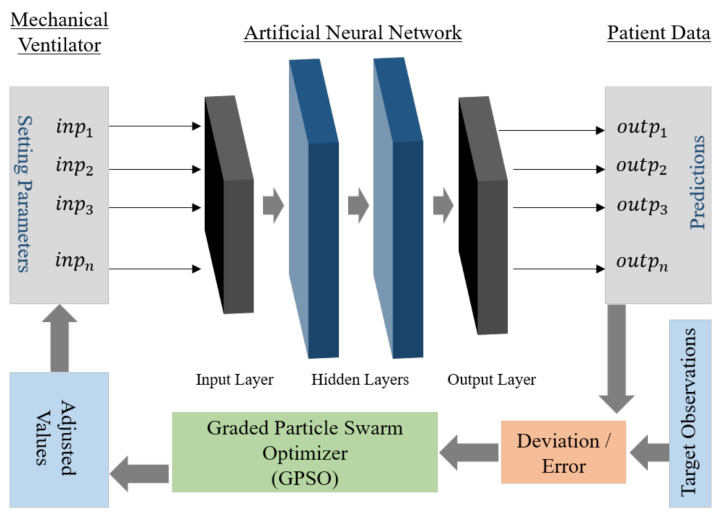
An outline of IANN for tuning mechanical ventilator parameters based on true observations. The mechanical ventilator inputs are, in the true sense, the outputs of the IANN. The GPSO in the loop compares the true observations with the predictions obtained from the ANN and makes adjustments to the inputs accordingly.

Artificial Neural Networks are statistical models for establishing non-linear relations between input and output data [20,21]. Hence, for a given set of observations with Y inputs and X outputs, an ANN can be trained to map a relationship between Y and X. Inverse mapping of neural networks (IANN) is a procedure conducted to obtain the correct input parameter values for a pre-trained ANN and a set of known outputs. An optimizer is applied to correct the input for the network iteratively until the output converges with the targets with minimal errors. This is widely used in many engineering applications, especially in manipulator inverse kinematics [22,23,24], sensor measurements [25], and structural integrity analysis [26]. Our current work used a population-based optimization algorithm in the feedback loop, which corrects and estimates the setting values given as inputs to the ANNs for the desired output (true observations). We applied a novel variant of particle swarm optimization (PSO) [27], the Graded Particle Swarm Optimizer (GPSO) proposed by Sanjay et al. [28]. An outline schematic of the inverse mapping model used in our current informatics model is presented in Figure 1.

Our current goal is to predict a range of possible values for mechanical ventilator settings for a given patient condition. Initially, a neural network is trained in the forward direction, i.e., the mechanical ventilator parameters as inputs and patient condition/observations as predictions or outputs. After deriving a well-trained network, it is used as a model for the system in the test phase. During this phase, the optimizer starts with a random value combination of ventilator settings as inputs to the ANN. The ANN generates a prediction for a patient condition that is compared against the true observations, and an error value is computed for the optimizer. The optimizer adjusts the inputs iteratively and terminates when the error converges or after meeting a certain criterion. As the modeled function (ANN) here is a highly non-linear and multi-dimensional function, a heuristic-based optimizer is preferred [29]. However, it must be noted that heuristic or search-based optimizer (like PSO [27]) may not generate the same value combination every time the process is initiated for the same patient condition. Hence, during the test phase, we provide the patient condition as input and expect the model to generate multiple possible combinations of ventilator parameter settings.

This is similar to an inverse kinematics problem, in which for reaching a certain position in space by the end effector, there can be multiple solutions for joint parameters [30]. Providing a range of values for a parameter can help support decision-making by the clinicians, instead of a single value generated by an ANN trained directly, i.e., with the patient condition as input and ventilator settings as output.

### 2.2. Graded Particle Swarm Optimizer (GPSO)

Ever since the introduction of Particle Swarm Optimization (PSO) by Kennedy and Eberhart [27], it has found many applications in the areas of control systems [31], signal processing [32,33], and machine learning [34,35,36], as well as where ever a complex, non-linear optimization is involved. PSO works on a real number space, and is quicker and easy to implement when compared with other swarm and computational intelligence (CI) techniques. However, the convergence at global optima is not guaranteed for all complex fitness functions, which also is true for the other CI techniques.

The general position and velocity update equations for each particle are as per Equations (2) and (3). Moreover, a widely used variant of PSO with an additional momentum factor ω is included in the equations.

(2) $$v^{t+1}\left(i\right)=\omega \cdot v^{t}\left(i\right)+r_{1}\cdot c_{1}\cdot \left(p_{best}^{t}\left(i\right)-x^{t}\left(i\right)\right)+r_{2}\cdot c_{2}\cdot \left(g_{best}^{t}-x^{t}\left(i\right)\right)$$

(3) $$x^{t+1}\left(i\right)=x^{t}\left(i\right)+v^{t+1}\left(i\right)$$

where $v_{i}\left(t\right)$ and $x_{i}\left(t\right)$ are the velocity and position values of the *i*-th particle during the *t*-th iteration, respectively. $r_{1},r_{2}$ are random numbers, and $c_{1},c_{2}$ are constants governing the personal and global influence on a particle. In every iteration, $g_{best}$ is computed for the position of the leader selected on the basis of the fitness value generated by a fitness function (best value), and $p_{best}$ is the best known position the particle visited until then. A higher $c_{2}$ value than $c_{1}$ has a greater influence of the leader on the particle than on its personal best.

PSO is known to get stuck at local optima applied on complex functions [37,38]. In order to improve the performance further, many variants were proposed over the past decade, which also focus on achieving quicker and higher probability of convergence in addition to achieving the optima [39,40]. Recent variants include a sub-population swarm strategy proposed by Wei Der Chang et al. [41], which involves individual groups searching for local optimum in a divided search space. Yen et al. [42] proposed particle exchange between sub-groups after a fixed number of iterations. Another recent variant, the club-based PSO [43], maintains a dynamic membership strategy by grouping and regrouping of particles based on their relative performance. We use a similar variant, the Gradient Particle Swarm Optimizer (GPSO) [28], in our current work.

The Graded Particle Swarm Optimizer (GPSO), in contrast to regular PSO, equally divides the swarm into ‘N-groups’, and a hierarchical group (N + 1) is generated with all the group leaders combined. The best in the group is called a universal leader. The GPSO is a modified form of PSO, where the hierarchical leader group’s effect is defined by the additional universal leader term in the velocity update, as shown in Equation (4).

(4) $$v^{t+1}\left(i\right)=\omega \cdot v^{t}\left(i\right)+r_{1}\cdot c_{1}\cdot \left(p_{best}^{t}\left(i\right)-x^{t}\left(i\right)\right)+r_{2}\cdot c_{2}\cdot \left(g_{best}^{t}\left(j\right)-x^{t}\left(i\right)\right)+r_{3}\cdot c_{3}\cdot \left(u_{best}^{t}-x^{t}\left(i\right)\right)$$

where $g_{best}^{t}\left(j\right)$ is the group best or group leader for group *j*; $u_{best}^{t}$ is the universal best; $r_{3}$ is a random number; and $c_{3}$ is a universal influence constant.

For a swarm with all the members in a single group, the group leader and universal leader turn out to be the same, merging the third and fourth terms in Equation (4). Similarly, for a swarm with n-groups and with a single member in each group, the third term turns into zero, with the group’s best being itself. In both these cases, Equation (4) reduces to the regular PSO condition in Equation (2).

**Figure 2:**
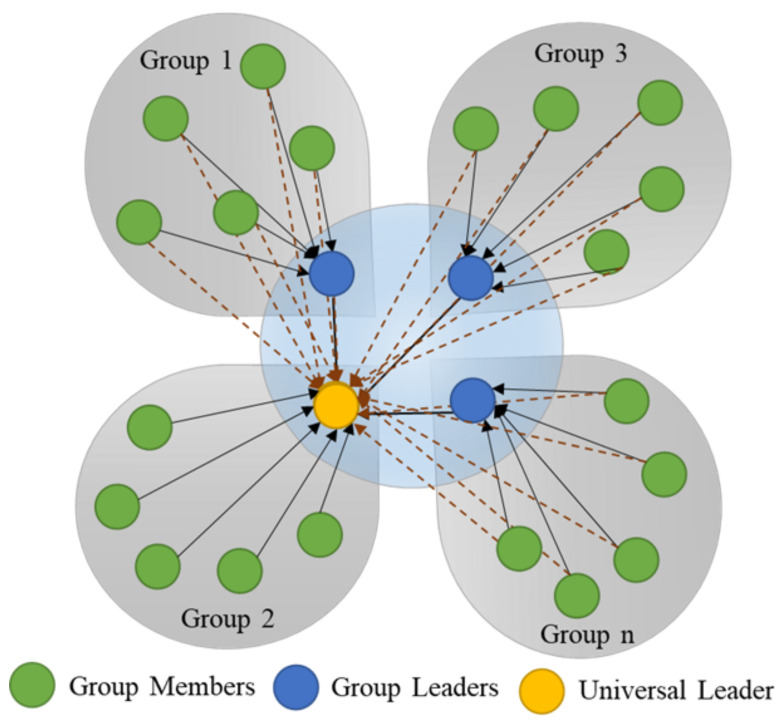
Organization of swarm in Graded Swarm Particle Swarm Optimizer. The swarm is divided into groups, and each group has a group leader. All the group leaders form a hierarchical group with a universal leader. All the particles/members in the group are influenced by the personal best, the group best, and the universal best vectors.

The GPSO algorithm was tested on standard benchmark functions [28], and it showed a higher probability of convergence, demonstrating its ability to avoid local optima. This is due to other groups’ influence through the universal leader in avoiding local optima when some individuals search for the nearest optimum. This is analogous to three forces acting on the particle, i.e., towards its personal experience, the group’s, best and the universal best. In short, when a group or a particle is trapped at a local optimum, the other better performing groups support its escape and hence avoid premature convergence. An illustration of groups and individuals with vectors acting in 2D on the individual particle is shown in Figure 2.

## 3. Data and Experiments

The experimental data of mechanical ventilation settings from 24 patients (canines—20 and felines—4) in the Small Animal Veterinary Teaching Hospital at the University of Georgia College of Veterinary Medicine were used to develop the current machine learning model.

Patients were classified as either having healthy or unhealthy lungs, and the data from ventilators along with their physical readings were collected over time. Six patients were classified as having healthy lungs and were ventilated to treat hypoventilation arising from disorders such as lower motor neuron disease, cervical spinal myelopathy, and drug toxicity; 18 of the patients were classified as having unhealthy lungs and were ventilated to treat hypoxemia caused by disorders such as congestive heart failure, pneumonia, and contusions. All the animal patients were anesthetized, most often with infusions of propofol and fentanyl, orotracheally intubated and mechanically ventilated using pressure-controlled ventilation in SIMV mode. All ventilation was conducted using the Respironics Esprit ventilator, and monitoring data were obtained using the NICO monitor (Novametrix Medical Systems Inc., Wallingford, CT, USA) [44,45,46].

The mixing of MV data from cats and dogs did not significantly alter the model performance. Aside from the body size (and subsequently smaller tidal volumes), physiologically, cats and dogs behave similarly on mechanical ventilators [7]. Moreover, some smaller dogs were also observed to have similar tidal volumes to those of cats. Further, the MV settings and desired outcome parameters are the same for both species.

### 3.1. Data Pruning and Segregation

Our current study’s data contained both physical observations and the data logged from the ventilator, which had definite time stamps. However, many missing data fields were identified in the patient observations as they were sporadically recorded. Hence, we used an ANN-based prediction for these missing data points. Multiple ANNs were trained using observations with complete data fields as inputs and the missing fields as output for the data. For example, an observation with a missing data point for $V_{ti}$ was predicted using an ANN trained with $V_{ti}$ as output and the remaining parameters other than $V_{ti}$ as inputs. The predicted missing data points were truncated to the nearest maximum or minimum values computed from the initial data sets. Furthermore, these pruned data were segregated into two sets for our inverse mapping model: the first set included the data for training, validating, and testing ANNs for the forward prediction; the second set was used for testing the inverse mapping process. The selection of data was completely random. A summary of data segregation is presented in Table 1.

### 3.2. Neural Network Modeling

In order to capture the dynamics of mechanical ventilation settings for different conditions/patients, we trained 200 artificial neural networks, and the top-ten best performers with the least total errors (training + validation + testing) ranging between $3.62\times 10^{3}$ and $4.0\times 10^{3}$ were selected for inverse mapping. Set pressure (SetP), peak input pressure (PIP), positive end expiratory pressure (PEEP), pressure support ventilation (PSV), respiratory rate (RR), and fraction of inspired oxygen (${\mathrm{FiO}}_{2}$) were considered as inputs, while tidal volume ($V_{ti}$), dynamic compliance ($C_{dyn}$), end tidal ${\mathrm{CO}}_{2}$ (${\mathrm{EtCO}}_{2}$), blood oxygen saturation (${\mathrm{SpO}}_{2}$), heart rate (HR), blood pressure (BP), and temperature were chosen as output parameters for the model. Each of these NNs was trained with the same data set (ANN generation), as presented in Table 1. However, the training, validation, and testing data sets were randomized for each neural network.

**Figure 3:**
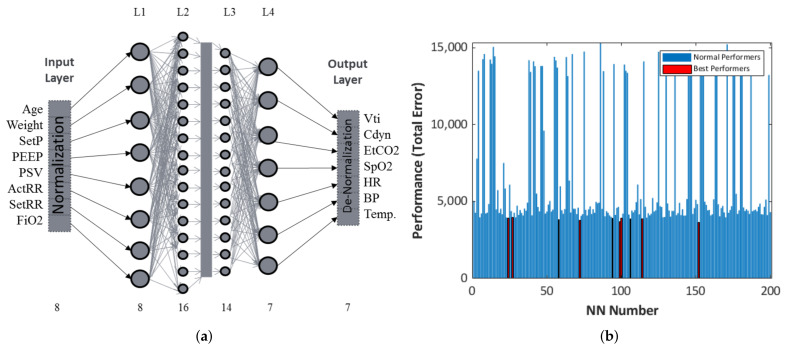
(**a**) Artificial neural network schema mapping ventilator parameter and patient outcomes. (**b**) Performance of different neural networks over 200 trials. Red represents the best performers that showed the lowest combined training, validation, and testing errors

NNs with four internal layers with a combination of 8, 16, 14, and 7 nodes in each layer were used in our current study. This configuration was arrived at after performance evaluation of different combinations of networks by comparing their total errors. The final schematic of NNs used for training is presented in Figure 3a. In addition, the top-ten best-performing NNs were selected based on low total errors. The total error data of all the trained networks are presented in the bar graph shown in Figure 3b.

**Figure 4:**
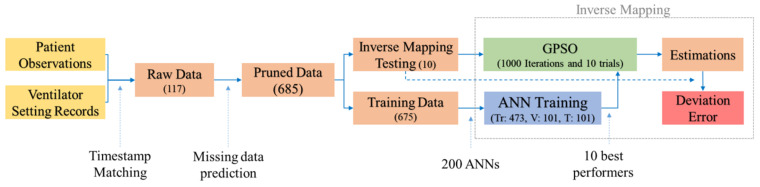
Block diagram shows a summary of data flow and operations carried out on the data at different stages.

Fitness computations are based on the outputs an ANN generates for a given input. The outputs normalized between their limits are compared with the targets, and an MSE as shown in Equation (5) is computed. IANN can be analyzed as a minimization problem where MSE in (5) is minimized by finding the right combinations of inputs for NNs. A summary of the process flow is presented in Figure 4, starting from data acquisition to the pruning, NN training, selection, and inverse mapping processes.

(5) $$MSE_{n}=\frac{1}{2}\sum {\left(OutputN_{i}-TargetN_{i}\right)}^{2}$$

where *i* represents the feature to be predicted.

### 3.3. Inverse Mapping Computations

An overview of the inverse mapping model is presented in Figure 4. This inverse mapping computation aims to estimate the inputs for a set of outputs with a pre-trained ANN. For more details, see Pidaparti et al. [26]. We carried out 1000 iterations for parameter estimation using the GPSO on each of the 100 trials, and the top performers in the swarm were selected in each trial. Test trials were performed for varying momentum, cognitive, group, and universal influence factors in the GPSO algorithm. After a preliminary analysis, we selected the parameters as shown in Table 2 for the current inverse mapping problem of identifying mechanical ventilation settings. Moreover, the trials were repeated on each of the top-ten best-performing neural networks, as shown in Figure 3b. The convergence criterion was the limit over the number of iterations or an MSE of less than 0.0001. While carrying out these iterations, two of the inputs—age and weight—were fixed, as these were known beforehand. This reduces the search dimensions of the swarm significantly and predicted the NN outputs more precisely. Moreover, the remaining parameters were kept within the known bounds in the data, i.e., the known minimum and maximum limits to be realistic. Any value out of these bounds was fixed to the nearest minimum or the maximum value of the parameter in every iteration.

We used the Deep Learning Toolbox in MATLAB 2021a (The MathWorks Inc., Natick, MA, USA) [47] in combination with programs developed for the GPSO. These simulations were run on a PC with Xeon 16 Cores and 128 GB RAM with 16 agents working in parallel.

## 4. Results and Discussion

**Figure 5:**
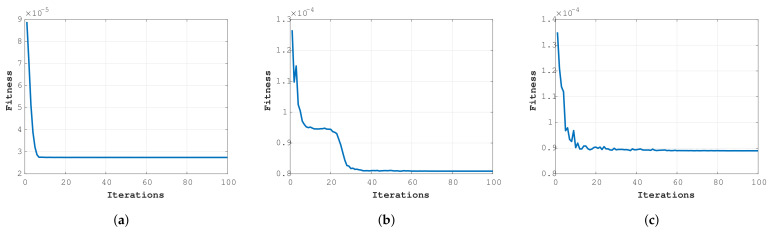
Performance graphs for (**a**) test 1, (**b**) test 2, (**c**), and test 3.

The typical fitness of the best trials over 1000 iterations for test data sets 1, 2, and 3 is presented in Figure 5. The graphs are for the best performers out of the 100 trials across the best NNs applied on 3 test data sets. Figure 5b best describes the way the GPSO algorithm works towards searching for the optimum from the sudden drop in fitness after a few iterations. This is because of the presence of multiple groups guiding each other, ensuring that the groups are not stuck local optima and thereby avoiding premature convergence.

**Figure 6:**
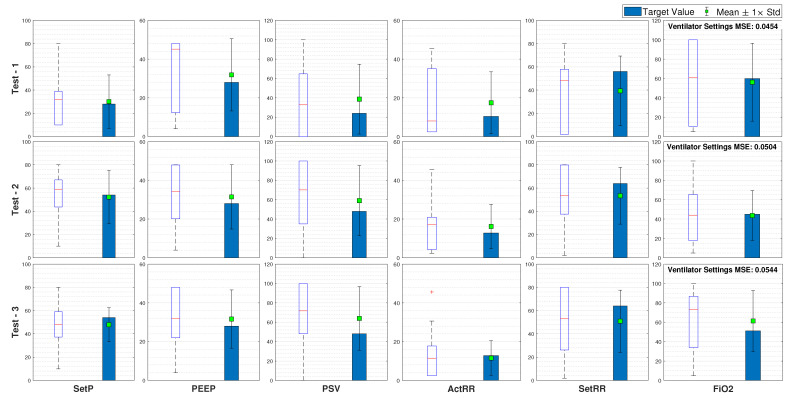
Statistics of results obtained on 10 test cases for 4 different ventilator settings.

Furthermore, on each of the ten data sets, we computed the maximum, minimum, average, and standard deviation for all the 100 trials, along with the best parameter combinations and their related MSEs. A summary of the results obtained for three test cases is presented in Figure 6.

The average parameter values computed across 100 trials and 10 networks are closer to the target values or the values set by the clinician for that patient. This shows that the inverse mapper approach presented in the paper converges to a set of inputs closely resembling the targets. The proposed GPSO also found other combinations of parameters, and, hence, we considered the average of input data across all the trials as possible values of the parameters.

In every trial, the model starts with random predictions within a range and keeps adjusting them until the output converges with the target outputs, leading to a different possible combination every time after termination. Hence, the range of values predicted may not be the desired parameter values. This is because the inverse mapper reduces the error between the NN outputs rather than the ventilator inputs, thus directly correlating to the trained NN’s efficiency. Hence, we chose multiple networks as candidates for the forward prediction/fitness evaluation to remove this possible bias.

## 5. Limitations of the Study

A limitation in the current study was the availability of a good amount of complete data for training the forward models. All the data in the present study were manually recorded, and these readings were taken at random time intervals (not continuous). Furthermore, the readings were specific to a patient’s condition, and the focus was mostly limited to particular parameters, leading to missing data fields. In our current study, the missing data were predicted by different NN models for completeness. This enhanced our data points to a significant number that was sufficient for our current studies. The current model can be applied to human data, and it is expected to demonstrate good confidence values when trained on larger amounts of data.

## 6. Conclusions

A machine learning model to estimate the mechanical ventilator parameters based on ANN and optimization was developed. After training the ANN with input/output data, the trained networks were used to estimate mechanical ventilation parameters through the inverse mapping technique. A novel GPSO algorithm based on our previous work [28] was adopted as an optimizer in inverse mapping computations. Extensive simulations were carried out to estimate the behavior of the GPSO applied in the feedback loop and for enhancing the model’s performance. The machine learning model was trained and tested using data from canine and feline patients at the University of Georgia College of Veterinary Medicine. Our model successfully generated a range of parameter values for the mechanical ventilation applied on test data, with the average prediction values over multiple trials close to the target values, even though the data sets’ availability was limited.

We observed that increasing the number of data points with high percentages of healthy patients can further stabilize the ANNs, as the data utilized for building our model were primarily from unhealthy patients because healthy patients are not usually observed on mechanical ventilators.

Overall, the developed machine learning model should be able to predict the mechanical ventilation parameters for various respiratory conditions once the relevant data are available. With the recent COVID-19 pandemic, the machine learning models proposed in this study should be of interest in predicting mechanical ventilation settings for patient survival given their respiratory health conditions.

## Figures and Tables

**Table 1:** Data segregation summary.

Process	Data Set	Number of Observations
Pruning	Original (complete)	117
	Pruned (Final)	685
ANN Modelling	Training	473
	Validation	101
	Testing	101
Inverse Mapping	Testing	10

**Table 2:** Parameters used in optimization.

Parameter	Value
Dimensions	8
Population	2000
Groups	50
Momentum (ω)	0.8
Personal/Cognitive Influence ($c_{1}$)	1.2
Group Leader’s Influence ($c_{2}$)	1.2
Universal Leader’s Influence ($c_{3}$)	1.2
Trials	100
Test ANNs	10
Termination	MSE < 0.01 Iterations = 100

## Data Availability

Data available on request, please contact the corresponding author at rmparti@uga.edu.
